# β-Asarone Rescues Pb-Induced Impairments of Spatial Memory and Synaptogenesis in Rats

**DOI:** 10.1371/journal.pone.0167401

**Published:** 2016-12-09

**Authors:** Qian-Qian Yang, Wei-Zhen Xue, Rong-Xin Zou, Yi Xu, Yang Du, Shuang Wang, Lai Xu, Yuan-Zhi Chen, Hui-Li Wang, Xiang-Tao Chen

**Affiliations:** 1 School of Food Science and Engineering, Hefei University of Technology, Hefei, Anhui, China; 2 School of Pharmacy, Anhui Medical University, Hefei, Anhui, China; University of Modena and Reggio Emilia, ITALY

## Abstract

Chronic lead (Pb) exposure causes cognitive deficits. This study aimed to explore the neuroprotective effect and mechanism of β-asarone, an active component from Chinese Herbs *Acorus tatarinowii* Schott, to alleviate impairments of spatial memory and synaptogenesis in Pb-exposed rats. Both Sprague-Dawley developmental rat pups and adult rats were used in the study. Developmental rat pups were exposed to Pb throughout the lactation period and β-asarone (10, 40mg kg^-1^, respectively) was given intraperitoneally from postnatal day 14 to 21. Also, the adult rats were exposed to Pb from embryo stage to 11 weeks old and β-asarone (2.5, 10, 40mg kg^-1^, respectively) was given from 9 to 11 weeks old. The level of β-asarone in brain tissue was measured by High Performance Liquid Chromatography. The Morris water maze test and Golgi-Cox staining method were used to assess spatial memory ability and synaptogenesis. The protein expression of NR2B subunit of NMDA receptor, Activity-regulated cytoskeleton-associated protein (Arc/Arg3.1) and Wnt family member 7A (Wnt7a) in hippocampus, as well as mRNA expression of Arc/Arg3.1 and Wnt7a, was also explored. We found that β-asarone could pass through the blood brain barrier quickly. And β-asarone effectively attenuated Pb-induced reduction of spine density in hippocampal CA1 and dentate gyrus areas in a dose-dependent manner both in developmental and adult rats, meanwhile the Pb-induced impairments of learning and memory were partially rescued. In addition, β-asarone effectively up-regulated the protein expression of NR2B, Arc and Wnt7a, as well as the mRNA levels of Arc/Arg3.1 and Wnt7a, which had been suppressed by Pb exposure. The results suggest the neuroprotective properties of β-asarone against Pb-induced memory impairments, and the effect is possibly through the regulation of synaptogenesis, which is mediated via Arc/Arg3.1 and Wnt pathway.

## Introduction

Lead (Pb) is a well-established environmental poison. It interferes with the development of the nervous system and the elevated blood lead levels in young children are associated with behavioral and cognitive deficits [1, 2]. Mechanically, Pb is a potent non-competitive antagonist of the N-methyl-D-aspartate (NMDA) receptor, which has been implicated as one of the principal target for Pb-induced deficits in long-term potentiation (LTP) and spatial learning process[3]. Also, Pb exposure during synaptogenesis alters NMDA receptor targeting via NMDA receptor inhibition [4].

β-asarone (cis-2,4,5-trimethoxy-1-allyl phenyl) is the major ingredient of the genus *Acorus* (e.g., *Acorus tatarinowii* Schott; ‘Sweet flag’) [5, 6]. *Acorus tatarinowii* has been used in oriental medicines to ameliorate learning and memory deficits [7–9]. For example, it is used as a component in some Chinese herbal formulas, such as *Kai-Xin-San* [10, 11] and *Chong-Myung-Tang* [12, 13], which have been applied to improve memory function. *Acorus tatarinowii* contains volatile oils, consisting mainly of α-asarone (8.8–13.7%) and β-asarone (63.2–81.2%) [7, 9]. β-asarone can easily pass through the blood brain barrier (BBB) [14] and substantial experimental evidence indicates that β-asarone is the active ingredient for attenuating learning and memory deficits [15–17]. Moreover, β-asarone could alleviate cognitive impairments in Parkinson’s disease [13], Alzheimer’s disease [18, 19], and neuroinflammatory [20], etc. Traditional use and clinical reports showed that β-asarone is effective for the treatment of learning and memory deficits, so we hypothesized that it may manage memory impairments following chronic Pb exposure.

Evidence suggests that spatial memory performance of rats in the Morris water maze (MWM) test is related to the level of granule cell neurogenesis [21]. Dendritic spines are major sites of excitatory synaptic transmission, and changes in their numbers and morphology have been associated with the deficits in synaptic plasticity and spatial learning [22]. Some proteins are involved in regulating the formation and structure of dendritic spines [23], such as Activity-regulated cytoskeleton-associated protein (Arc/Arg3.1) [24] and Wnt family member 7A (Wnt7a) [25].

In the present study, we aimed to assess β-asarone’s effects on spatial memory and synaptogenesis in Pb-exposed rats. We found that β-asarone rescued the Pb-induced spatial memory deficits both in development and adult rats, possibly through altering NR2B subunit of NMDA receptor, protein and mRNA expression of Arc/Arg3.1 and Wnt7a.

## Materials and Methods

### β-asarone preparation

**Fig 1:**
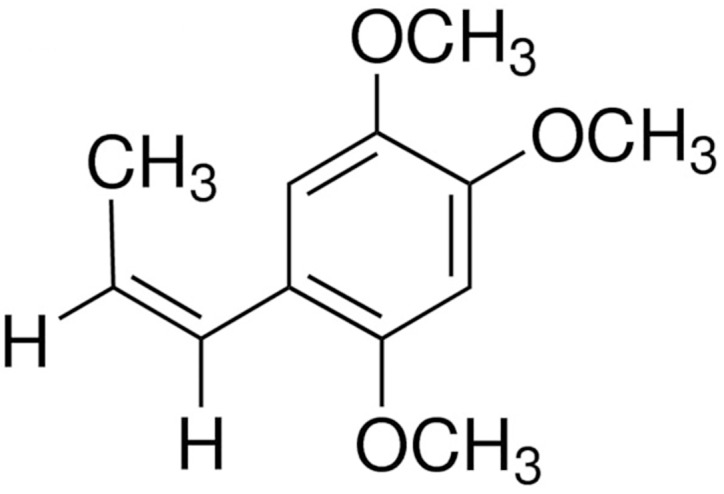
The chemical structure of β-asarone.

β-asarone was obtained from Sigma-Aldrich Co. LLC (CAS: 5273-86-9), which was isolated from the extract of Acorus gramineus using various chromatographic procedures (for its structure, see Fig 1). It is a fat-soluble substance with a small molecular weight, and was made by dissolving in 2% Tween-80 (Sinopharm Chemical Reagent Co., Ltd).

### Animals and experimental design

Sprague–Dawley rats were supplied by the Laboratory Animal Center, Anhui Medical University, P.R. China. Rats were individually housed in a temperature (20±3°C) and humidity (50±10%) controlled environment on a 12 hrs-12 hrs light-dark cycle with free access to food and water. This study was carried out in strict accordance with the recommendations in the Guide for the Care and Use of Laboratory Animals of the National Institutes of Health and was approved by the Institutional Animal Care and Use Committee of Anhui Medical University, P.R. China. The movements, food intake, body weight and body temperature of animals were monitored twice daily to determine their health and activity levels, including weekends and holidays. There was no severe illness and unintended death of animals during this study. All surgery was performed under carbon dioxide anesthesia, and all efforts were made to minimize animal suffering.

Due to potential toxic effects of β-asarone reported [26], first we investigated the dosage of β-asarone administration to observe the potential toxicity. Body weight and physical condition are the key considerations when the rats were given intraperitoneal injection of β-asarone. Accordingly, 80mg kg^-1^day^-1^ of β-asarone led to weakness, mental fatigue, body weight loss and even some death after 7 days of injection, while 40mg kg^-1^ day^-1^ injection did not produce significant adverse effects. So we chose 40mg kg^-1^day^-1^ of β-asarone as higher dosage in our experimental design. The developmental rats were randomly divided into six groups as follows: (1) control group; (2) control+β-asarone (10mg kg^-1^day^-1^); (3) control+β-asarone (40mg kg^-1^day^-1^); (4) Pb-exposed; (5) Pb+β-asarone (10mg kg^-1^day^-1^); (6) Pb+β-asarone (40mg kg^-1^day^-1^) (n = 8 in each group). The method for chronic Pb exposure was referred to the previous studies [25]. The day of birth was considered as postnatal day (PND) 1. The Pb-exposed pups acquired Pb via milk of dams during lactation period indirectly, whose drinking water contained Pb (250ppm PbAc, 30ml per day) and then directly after weaning. The control dams received distilled water. The β-asarone treated pups were received a daily intraperitoneal injection of β-asarone from PND 14 to PND 21. This period is a key stage of vulnerability for the developing nervous system in rodents [27]. In all six groups, equal numbers of female and male pups were used. Only one pup per litter was selected for the experiment.

For the adult rat test, rats of 9 weeks old were used. The adult rats were randomly divided into five groups as follows: (1) control group; (2) Pb-exposed; (3) Pb+β-asarone (2.5mg kg^-1^day^-1^); (4) Pb+β-asarone (10mg kg^-1^day^-1^); (5) Pb+β-asarone (40mg kg^-1^day^-1^) (n = 7 in each group). The Pb-exposed adult rats acquired Pb from embryo stage to 11 weeks old via drinking water which containing Pb (250ppm PbAc, 30ml per day), and the β-asarone treated rat were received a daily intraperitoneal injection of β-asarone from 9 to 11 weeks old.

After the last administration of β-asarone, the animals were subjected to the MWM test and then killed under deep anesthesia with CO_2_ one day after the last MWM test. Brain tissues were collected for subsequent experiments.

### Measurement of β-asarone in brain tissue

Measurement of β-asarone in brain tissues was performed by High Performance Liquid Chromatography (HPLC) analysis. 1g of brain tissues were grinded in mortar with 1ml of cold phosphate-buffered saline (pH 7.4). The brain tissue was transferred into a tube to homogenize by sonication (50W×15s) on ice. β-asarone was extracted by 1.5ml HPLC-grade hexane (Sinopharm Chemical Reagent Co., Ltd), and then the organic layer was filtered. β-asarone was separated under isocratic condition using 70% HPLC-grade methanol (Sinopharm Chemical Reagent Co., Ltd) in ultrapure water and a column (Inertsil ODS-3, 5μm, 4×250mm). The absorbance of the organic layer was measured at 257nm. Compound identification and analysis calibration were based on use of β-asarone as external standards.

### Golgi-Cox staining and spine density assay

Rats were anesthetized with CO_2_ and quickly decapitated. The brains were longitudinally cut into two halves. One hemisphere was processed for morphological staining and the other hemisphere was used to examine the expression of specific proteins. The Golgi-Cox staining was applied with minor modification as described by Hu et al [25]. Briefly, brains stored in dark place for 2 days (37°C) in Golgi-Cox solution were sectioned at 200μm in the 6% sucrose with a vibratome (VT1000S, Leica, Germany). All sections were collected on 2% gelatin-coated slides. Then slices were stained with ammonia for 60 mins, washed with water for 3 times, followed by Kodak Film Fix for 30 mins, and then washed with water, dehydrated, cleared, and mounted using a resinous medium. The pyramidal neurons in hippocampal CA1 and Dentate gyrus (DG) regions were imaged with a widefield microscope (Eclipse 80i, Nikon) using a 40x objective.

Then, spine densities were calculated as mean numbers of spines per 10μm per dendrite per neuron in individual rat per group. The spines counted in the present study were on 2, 3 stretches of the secondary dendrite about 20mm in length.

### MWM test

The MWM experiments were performed in a circular pool with a diameter of 160cm and a depth of 70cm. It filled to a depth of 40cm with opaque water by addition of caramel coloring, keeping a temperature of 23±1°C. The rats were gently put in the water facing the wall of pool. Each rat was trained for four trials daily for 5 days to find the hidden platform. When found the platform, it had 30 secs staying on it. If failed to reach the platform within 60 secs, it was guided and allowed to remain on the platform for the same period of time. The platform was removed on the sixth day, then each rat was afforded 60 secs for probe trial. Learning was assessed by measuring the latency to find the platform. For characterization of memory, the number of potential platform crossings and time spent in the target quadrant during the probe trial were assessed.

### Western blot analysis

Hippocampal tissues were homogenized and dissolved in the ice lysis-buffer containing a cocktail of protein phosphatase and protease inhibitors (21μg/ml aprotinin, 0.5μg/ml leupetin, 4.9mM MgCl_2_, 1mM sodium-Meta-vanandante, 1% Triton X-100 and 1mM PMSF) to avoid dephosphorylation and degradation of proteins. The samples were centrifuged at 14000rpm at 4°C for 7 mins. The total protein of supernatant was quantified using the Bicinchoninic acid protein assay (Beyotime Biotechnology). 30μg of proteins were resolved using 12% SDS-PAGE, transferred onto PVDF membrane (Merck Millipore). The blot was probed with primary antibodies of NR2B, Arc/Arg3.1, Wnt7a and GAPDH (Abcam, Inc.). Proteins were incubated with secondary antiboby and visualized using the electrochemiluminescence method. All the results were normalized against GAPDH. Each target protein was performed 3 times.

### Semi-quantitative PCR

Total RNA of hippocampus extracted by AxyPrep Multisource Total RNA Miniprep Kit (Axygen, USA) was used for cDNA transcription with cDNA Transcription Kit (TransGen, China). Semi-quantitative PCR was performed on a S1000 Thermal Cycler (Bio-Rad, USA) using primers pairs listed in Table 1. The results were normalized against β-actin as an internal control. Each target gene was performed 3 times.

**Table 1:** Primer sequences.

Gene	primers
Arc	F 5’-GACTACACTGTTAGCCCCTATGC-3’
	R 5’-TCTTCACCGAGCCCTGTTTG-3’
Wnt7a	F 5’-CCAGTTCAAACCTCGCCATTAG-3’
	R 5’-AAGGAATCAGCCATACATCGTG-3’
β-actin	F 5’-CCTGAAGTACCCCATTGAAC-3’
	R 5’-GAGGTCTTTACGGATGTCAAC-3’

### Statistical analysis

All data were expressed as mean ± SEM. One-way ANOVA was applied to analyze the data of HPLC, dendritic spine density, Western blot protein assay and Semi-quantitative PCR assay. Two-way ANOVA was used to the data of training in MWM test. Difference between experiment groups was tested by Fisher’s protected least significant difference (PLSD) with 95% confidence. *P*<0.05 indicates a significance difference.

## Results

### β-asarone concentration in brain tissue

**Fig 2:**
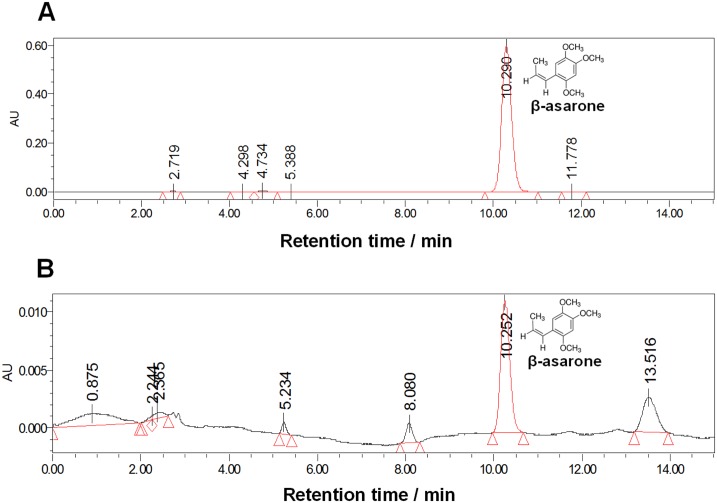
Measurements of β-asarone in brain tissue with HPLC analysis. (A) The chromatogram of standard β-asarone. (B) The chromatogram of β-asarone extracted from brain tissues of developmental rats one hour after once intraperitoneal injection of β-asarone (40mg kg^-1^).

We first investigated whether β-asarone could pass through the BBB. The β-asarone in brain tissues was measured with HPLC analysis using β-asarone as external standards for calibration (Fig 2A). The result showed that rat pups exhibited 6.769±0.187μg g^-1^ of β-asarone in the brain one hour after once intraperitoneal injection of β-asarone (40mg kg^-1^) (Fig 2B). The result was consistent with previous study [14], which suggests that the β-asarone could penetrate the BBB and be very rapidly absorbed in brain tissues.

### β-asarone treatments reversed Pb-induced decrease of dendritic spine density in hippocampal CA1 and DG areas of developmental rats

**Fig 3:**
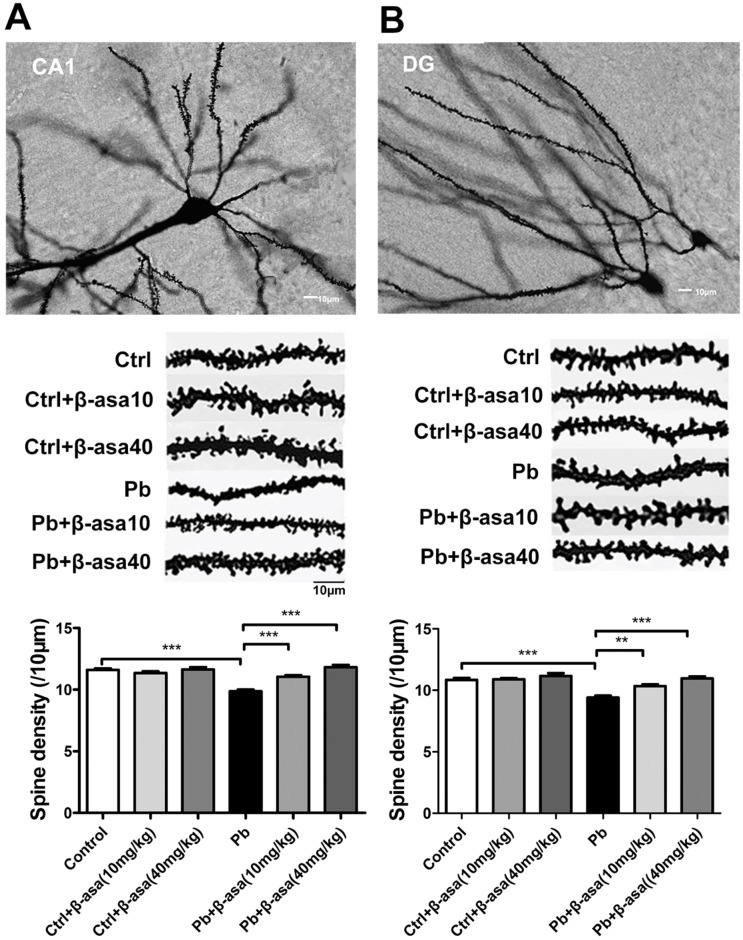
Effects of β-asarone on dendritic spine density in Pb-exposed developmental rats. (A) CA1 area; (B) DG area. The top: Representative Golgi-Cox staining of dendritic arborization and dendritic spine density. The middle: Representative dendritic shaft with spines of hippocampal neurons. Scale bar = 10μm. The bottom: Histogram reports the quantification of spine density (10μm). (**P<0.01, ***P<0.001, n = 8 per group; β-asa: β-asarone, the same in the following Figs).

Our recent work showed that Pb exposure significantly decreased the spine density in both 14 and 21 days old pups [25], we wonder whether the addition of β-asarone could reverse this impairment at very early developmental hippocampus. It is showed that, by Golgi-Cox staining process, Pb exposure significantly decreased the spine density both in CA1 (Fig 3A) and DG (Fig 3B) areas, while β-asarone administration significantly recovered the drop in spine density in a dose-dependent manner. This result indicated that β-asarone treatments repaired Pb-induced impairments in spine formation in developmental hippocampus.

### β-asarone treatments dose-dependently attenuated Pb-induced memory deficits in adult rats

**Fig 4:**
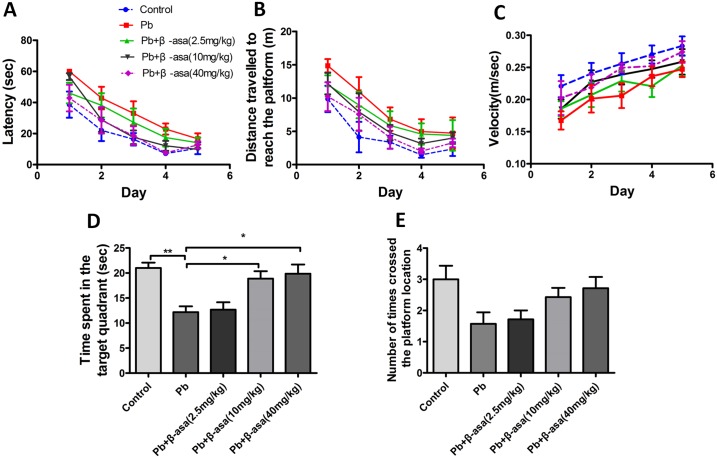
Effects of β-asarone on MWM tests in Pb-exposed adult rats. (A) Latency; (B) Distance travelled to reach the platform; (C) Swimming velocity during MWM tests; (D) Time spent in target quadrant; (E) Number of times crossed the platform in probe trial. (*P<0.05, **P<0.01, n = 7 per group).

Then we asked whether β-asarone treatment effectively attenuated memory deficit in Pb-exposed rats. MWM test was employed to evaluate the ability of spatial learning and memory in adult rats. The Pb exposure and training days significantly affected the average latency (*F*_(1, 70)_ = 66.033, *P*<0.001; *F*_(4, 70)_ = 16.589, *P*<0.001) (Fig 4A) and distance (*F*_(1, 70)_ = 34.503, *P*<0.001; *F*_(4, 70)_ = 16.507, *P*<0.001) (Fig 4B) travelled to the hidden platform, while the damage induced by Pb exposure could be partly rescued by β-asarone treatment. Two-way ANOVA analysis found that β-asarone treatment or training days remarkably decreased latency (*F*_(3,140)_ = 14.125, *P*<0.001; *F*_(4,140)_ = 24.460, *P*<0.001) (Fig 4A) and distance (*F*_(3,140)_ = 3.782, *P*<0.05; *F*_(4,140)_ = 21.893, *P*<0.001) (Fig 4B) travelled to the hidden platform. The training days significantly affected the swim speed (velocity) (*F*_(4, 70)_ = 6.339, *P*<0.001; *F*_(1, 70)_ = 0.436, *P* = 0.511), but Pb failed (Fig 4C). There are no significant changes observed in distance (*F*_(12,140)_ = 1.265, *P* = 0.248), latency (*F*_(12,140)_ = 0.901, *P* = 0.548) and velocity (*F*_(12,140)_ = 0.654, *P* = 0.791) following interactions of β-asarone treatment×training days.

Probe tests showed that Pb exposure decreased the time spent in target quadrant (control, 21.01±1.06; Pb, 12.19±1.16, *P*<0.01); and higher dosage of β-asarone (10mg kg^-1^day^-1^, 40mg kg^-1^day^-1^) significantly increased the time compared with Pb-exposed group, but lower dosage of β-asarone (2.5mg kg^-1^day^-1^) failed (Pb, 12.19±1.16; Pb+β-asarone 2.5mg kg^-1^day^-1^, 12.67±1.49, *P*>0.05; Pb+β-asarone 10mg kg^-1^day^-1^, 18.89±1.48, *P*<0.05; Pb+β-asarone 40mg kg^-1^day^-1^, 19.86±1.85, *P*<0.05) (Fig 4D). There was no significant difference between all groups of the times crossed the platform (Fig 4E). Collectively, β-asarone treatments retrieved the Pb-induced spatial memory deficits in a dose-dependent manner.

### β-asarone treatments increased dendritic spine density in CA1 and DG areas in Pb-exposed adult rats

**Fig 5:**
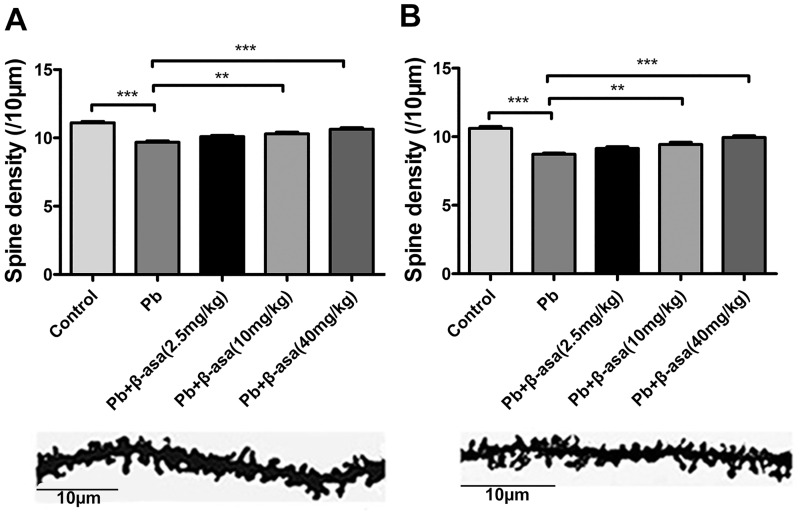
Effects of β-asarone on dendritic spine density of hippocampus in Pb-exposed adult rats. (A) CA1 area; (B) DG area. Histogram reports the quantification of spine density. (**P<0.01, ***P<0.001, n = 8 per group).

Given β-asarone effectively rescued the spatial memory deficits in Pb-exposed adult rats, we wonder whether it accompanied with the spine morphological changes. The results showed that Pb exposure significantly decreased the dendritic spine density both in CA1 and DG areas, while β-asarone administration recovers the reduction induced by Pb exposure (Fig 5). It suggested that β-asarone dose-dependently repaired the Pb-induced deficits of dendritic spines formation in both CA1 and DG areas in adult rats. Taken together, β-asarone treatments showed similar effect in both developmental and adult rats.

### β-asarone treatments up-regulated NR2B, Arc/Arg3.1 and Wnt7a protein expression in Pb-exposed adult rats

**Fig 6:**
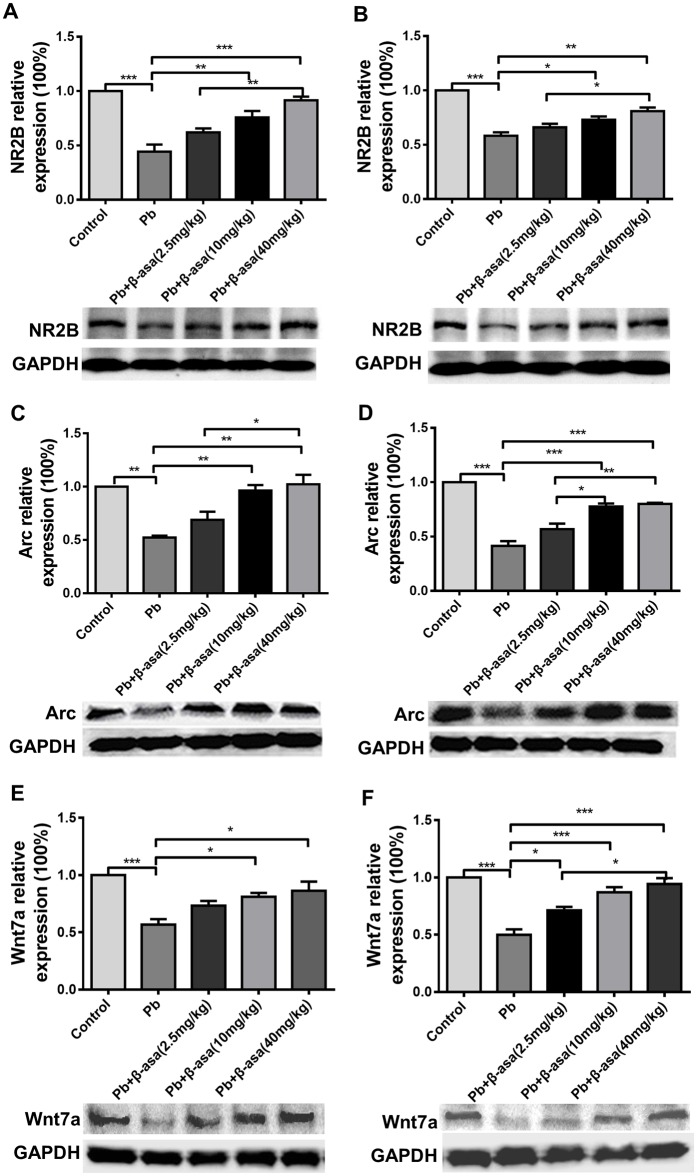
Effects of β-asarone on the relevant protein expression in Pb-exposed adult rats. Western blot analysis of the protein expression in hippocampus. Histogram represents densitometric analysis of blots from 3 independent experiments (Mean±SEM). Left: CA1 area; right: DG area. (A) and (B) NR2B; (C) and (D) Arc/Arg3.1; (E) and (F) Wnt7a. (*P<0.05, **P<0.01, ***P<0.001, n = 8 per group).

NR2B and Arc/Arg3.1 are linked to synaptic plasticity and memory. Western blot assay results showed that NR2B expression reduced by 41% after Pb exposure in CA1 area, while β-asarone supplement restored the reduction in a dose-dependent manner (Pb+β-asarone 2.5mg kg^-1^day^-1^, 12.7%; Pb+β-asarone 10mg kg^-1^day^-1^, 30%; Pb+β-asarone 40mg kg^-1^day^-1^, 41.2%) (Fig 6A). Arc/Arg3.1 expression decreased by 47.7% after Pb exposure in CA1 area, while β-asarone treatment recovered the decrease dose-dependently (Pb+β-asarone 2.5mg kg^-1^day^-1^, 16.7%; Pb+β-asarone 10mg kg^-1^day^-1^, 44%; Pb+β-asarone 40mg kg^-1^day^-1^, 50.1%) (Fig 6C). The expression of NR2B (Fig 6B) and Arc/Arg3.1 (Fig 6D) in DG area showed the same trend as that in CA1 area.

The Wnt signaling pathway is one of the central morphogenic signaling pathways regulating early neuronal development and Wnt7a preferentially stimulates excitatory dendritic spine formation [28]. Our recent work showed Pb exposure resulted in synapse damage through Wnt7a pathway [25]. In the present study, the relative expression of Wnt7a in hippocampal CA1 area of Pb-exposed rats significantly decreased by 43.2%, which is consistent with our previous results, while β-asarone treatment dose-dependently recovered the decreasing (Pb+β-asarone 2.5mg kg^-1^day^-1^, 16.5%; Pb+β-asarone 10mg kg^-1^day^-1^, 24.2%; Pb+β-asarone 40mg kg^-1^day^-1^, 29.6%) (Fig 6E). In DG area, we found Pb exposure decreased Wnt7a by 50% compared with control group, while β-asarone treatment recovered the decreasing in a dose-dependent manner (Pb+β-asarone 2.5mg kg^-1^day^-1^, 21.3%; Pb+β-asarone 10mg kg^-1^day^-1^, 37%; Pb+β-asarone 40mg kg^-1^day^-1^, 44.3%) (Fig 6F).

**Fig 7:**
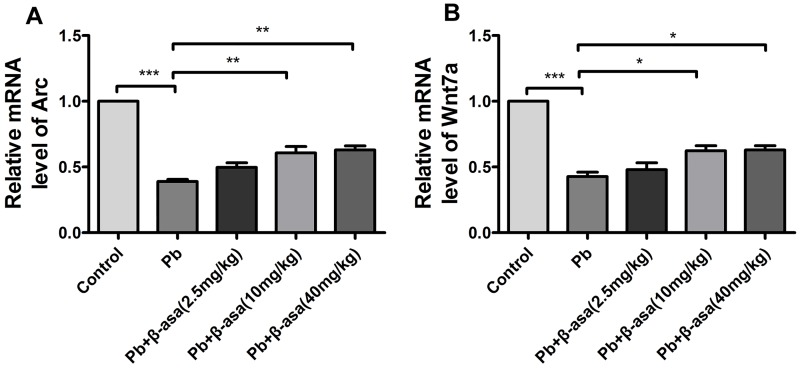
Effects of β-asarone on transcription level of Arc/Arg3.1 and Wnt7a in Pb-exposed adult rats. RT-PCR exhibits the transcription level. (A) Arc/Arg3.1; (B) Wnt7a. Histograms show densitometric analyses of PCR from 3 independent experiments (Mean±SEM). (*P<0.05, **P<0.01, ***P<0.001, n = 8 per group).

We next assessed the transcription levels of Arc/Arg3.1 and Wnt7a in hippocampus. Pb exposure significantly decreased Arc/Arg3.1 and Wnt7a mRNA level (61% and 57%) (Fig 7). And β-asarone significantly increased the target genes transcription level (for Arc/Arg3.1 mRNA, Pb+β-asarone 2.5mg kg^-1^day^-1^, 10.7%, Pb+β-asarone 10mg kg^-1^day^-1^, 22%, Pb+β-asarone 40mg kg^-1^day^-1^, 24%; for Wnt7a mRNA, Pb+β-asarone 2.5mg kg^-1^day^-1^, 5%, Pb+β-asarone 10mg kg^-1^day^-1^, 19%, Pb+β-asarone 40mg kg^-1^day^-1^, 20%). These results suggested that β-asarone has a contribution to the up-regulation of NR2B, Arc/Arg3.1 and Wnt7a, which may directly or indirectly protect the brain against Pb-induced impairments.

## Discussion

The present study showed that β-asarone ameliorated the memory impairment induced by Pb exposure in a dose-dependent manner. In details, we found that β-asarone rescued the Pb-induced spatial memory deficits; retrieved the Pb-induced decrease of dendritic spine density in hippocampal pyramidal neurons both in developmental and adult rats; up-regulated the expression of NR2B subunit of the NMDA receptor, immediate-early gene Arc/Arg3.1 and Wnt7a, an inducer of Wnt pathway.

Dendritic spines arise as small protrusions from the dendritic shaft of various types of neuron and receive inputs from excitatory axons. They contain neurotransmitter receptors, organelles, and signaling systems essential for synaptic function and plasticity [29]. LTP promotes new spine formation [30, 31]. Dendritic spine density directly reflects the number of excitatory inputs to pyramidal neurons, and reduced dendritic spine density has been associated with memory deficits [22]. Our previous work showed that Pb exposure significantly decreased the hippocampal LTP [32] as well as the spine density of pyramidal neurons in hippocampus [25]. In this study, β-asarone rescued the decrease in spine density. To some extent, this trend is consistent with the neuroprotective effects of β-asarone against the Pb-induced spatial memory deficits. Moreover, Pb-induced decrease of spine formation can be observed as early as PND 14 [25]. In this study, β-asarone retrieved the decrease in spine density in both early developmental and adult hippocampus. Collectively, it implies that β-asarone could improve spine formation and maturity to refine synaptic efficacy in Pb-exposed rats.

The NMDA receptor is the predominant molecular device for controlling synaptic plasticity and memory function. NMDA receptor is composed of NR1 and NR2A-D subunits and its activation requires glutamate and glycine. Glycine-binding site is in the NR1 subunit and the glutamate-binding domain is present in NR2B subunit. Early in development, NMDA receptor complexes in nascent synapses are primarily of the NR1/NR2B type [33]. Previous studies have found that Pb exposure during synaptogenesis alters NMDA receptor synapse development via NMDA receptor inhibition [4]. And Pb impairs NMDA receptor-dependent LTP induction in hippocampus in rats [34] and down-regulates the expression of NR2A and NR2B subunits [35]. In this study, we found that the Pb-induced down-regulation of NR2B subunits in hippocampus were partially reversed with β-asarone.

It should be noted that some studies reported Pb exposure during synaptogenesis in cultured hippocampal neurons resulted in a decrease in NR2A-containing NMDA receptors, while a increase in NR2B-containing NMDA receptors [4, 36]. It seems in conflict with our present result. In fact, during brain development, there is a shift from NR2B- to NR2A-containing NMDA receptors. Those studies focused on the altered composition of NMDA receptors, and it is possibly that the Pb exposure changed the proportion of the NR2B subunit, instead of the total number of it, suggesting Pb exposure probably impairs or delays the developmental switch during synaptogenesis. Our present study determined the NR2B protein level before and after Pb exposure and β-asarone treatment. Actually, both up- and/or down-regulation of NR2A/2B can plausibly disrupt the normal development of temporal processing, and β-asarone may rescue the impairment in development and thereby improve memory.

Some proteins are involved in regulating the formation and structure of dendritic spines [23], such as Arc/Arg3.1 [24] and Wnt7a [25]. Arc/Arg3.1, an immediate-early gene, plays an important role in synaptic plasticity [37, 38], α-amino-3-hydroxy-5-methyl-4-isoxazole propionate(AMPA) receptor trafficking [39] and neuro-behavior activity. NMDA receptor activation increases Arc/Arg3.1 levels [40] and sustained Arc/Arg3.1 synthesis has been implicated in LTP consolidation and spine formation [41]. We observed a significant correlation between the increases in NR2B and Arc/Arg3.1 expression levels following β-asarone treatment.

NMDA receptor activation also leads to Wnt release and β-catenin accumulation in hippocampus [42]. Wnt can promote the presynaptic assembly [43]. Wnt and Brain-derived neurotrophic factor (BDNF) cooperatively increase dendritic spine formation [44]. Inhibition of Wnt signaling leads to decrease in arbor size of spine and is linked with neurodegenerative disease [45]. As an inducer of Wnt signaling pathway, Wnt7a can increase neurotransmitter release in hippocampal synapses by increasing the frequency of miniature excitatory post-synaptic currents (mEPSC) [46].

Our recent work showed that Pb exposure significantly decreased the spine density, accompanied with down-regulated Wnt7a expression [25]. In the present study, β-asarone restored the Pb-induced decrease of Arc/Arg3.1 and Wnt7a transcription and expression, as well as the reduction of spine density in hippocampus. In addition to our findings, β-asarone has been reported to reverse decreased BDNF and promote hippocampal neurogenesis in chronic unpredictable mild stress exposed rats [47]. Besides, β-asarone activated extracellular signal-regulated kinase (ERK), a critical kinase cascades for neurogenesis, to promote proliferation and self-renewal in neural progenitor cell [48]. BDNF can trigger the Wnt/β-catenin signaling pathway [49]. It could mobilize synaptic vesicles and enhance synapse formation by disrupting cadherin–β-catenin interactions [50]. It can regulate axon morphogenesis by influencing the phosphorylation state of β-catenin [51]. Moreover, β-catenin signaling can also activate the ERK signaling pathway [52]. For example, axin, a negative regulator of Wnt/β-catenin signaling, can inhibit ERK Pathway [53]. And the Wnt antagonist Dkk1 and the β-catenin degradation stimulator Axin2 can abolish mechanical stimulation-induced ERK nuclear translocation [54]. These reports together with our results suggest BDNF/Wnt signaling pathway might represent a key target for various neurogenesis agents, including β-asarone.

It has been previously established that the synaptic activation of NMDA receptors leads to PKA-dependent increase of Arc/Arg3.1 expression [55, 56]. Since Pb exposure down-regulated expressions of NR2B subunits of NMDA receptors, while β-asarone reversed the drop accompanied with the decrease of Arc/Arg3.1 and Wnt7a. It might be suggested that β-asarone may function by up-regulating of the NR2B, BDNF/Wnt, and ERK signaling leading to increase in Arc/Arg3.1 expression. Although the exact molecular targets of β-asarone in promoting neurogenesis are still unclear, our study provided preliminary hints for their underlying mechanisms.

In conclusion, the present study demonstrated that β-asarone has neuroprotective properties against the Pb-induced impairments of spatial memory as well as dendritic spine morphological alteration. And the effect is possibly through the regulation of synaptogenesis, which is mediated via Arc and Wnt pathway.

## Supporting Information

S1 File Experimental data of HPLC, MWM, qRT-PCR, Spine dinsity, Western-blot. (ZIP)
