# Supplementary material for: β-Asarone Rescues Pb-Induced Impairments of Spatial Memory and Synaptogenesis in Rats
Source: PLoS One. 2016 Dec 9;11(12):e0167401. doi: 10.1371/journal.pone.0167401 (PMC5147873; doi:10.1371/journal.pone.0167401)
Supplement: S1 File — (ZIP) [file pone.0167401.s001.zip › data set/HPLC resuls.doc]

â-asarone 4ìL

	retention time	Peak area	Peak area(%)	Peak height	
1	0.267	18022	0.46	945	
2	0.717	41537	1.07	1811	
3	2.717	19265	0.50	2203	
4	3.442	149422	3.85	2606	
5	4.190	75485	1.94	2441	
6	4.760	29411	0.76	3107	
7	5.315	108227	2.79	2715	
8	10.352	3391400	87.37	234450	
9	12.893	48845	1.26	631	
â-asarone 8ìL

	retention time	Peak area	Peak area(%)	Peak height	
1	3.092	19410	0.46	442	
2	4.298	5080	0.12	420	
3	4.743	20515	0.48	2698	
4	6.184	50863	1.19	1057	
5	10.328	4155192	97.48	286551	
6	11.793	11713	0.27	806	

â-asarone 12ìL

	retention time	Peak area	Peak area(%)	Peak height	
1	0.025	1538	0.02	284	
2	2.708	4985	0.07	670	
3	3.022	21836	0.32	763	
4	4.296	33102	0.48	1451	
5	4.737	40322	0.58	4335	
6	5.990	2201	0.03	369	
7	10.315	6776833	98.26	438221	
8	11.825	15785	0.23	1001	


â-asarone 16ìL

	retention time	Peak area	Peak area(%)	Peak height	
1	0.024	1175	0.01	260	
2	2.713	30624	0.37	1188	
3	3.088	48059	0.58	998	
4	4.296	11035	0.13	804	
5	4.736	42334	0.51	4867	
6	5.379	4599	0.06	350	
7	10.303	8102350	98.11	529448	
8	11.793	18302	0.22	1194	

â-asarone 20ìL

	retention time	Peak area	Peak area(%)	Peak height	
1	2.719	12652	0.13	1439	
2	4.298	21303	0.22	1255	
3	4.734	50930	0.53	5406	
4	5.388	144682	1.50	1197	
5	10.290	9423467	97.41	600324	
6	11.778	21456	0.22	1355	

â-asarone  sample  20ìL

	retention time	Peak area	Peak area(%)	Peak height	
1	0.875	69353	21.11	1056	
2	2.244	1993	0.61	333	
3	2.365	7655	2.33	524	
4	5.234	7420	2.26	1072	
5	8.080	17593	5.35	1693	
6	10.252	159723	48.61	11386	
7	13.516	64840	19.73	3032	
					

â-asarone sample 20ìL-1

	retention time	Peak area	Peak area(%)	Peak height	
1	1.739	37621	9.14	501	
2	2.825	22270	5.41	1229	
3	4.891	7657	1.86	502	
4	5.175	6951	1.69	1009	
5	6.039	4053	0.98	490	
6	6.399	42949	10.44	1180	
7	7.943	43214	10.50	2730	
8	10.021	152652	37.09	11044	
9	11.448	16823	4.09	596	
10	13.492	77337	18.79	3336	

â-asarone sample 40ìL-1


	retention time	Peak area	Peak area(%)	Peak height	
1	0.321	32028	2.66	1314	
2	2.688	7870	0.65	1171	
3	2.803	9425	0.78	1609	
4	3.963	13979	1.16	404	
5	5.091	29993	2.49	2958	
6	5.458	12954	1.08	1110	
7	5.841	35893	2.99	1438	
8	6.248	70228	5.84	2199	
9	7.212	55612	4.63	2188	
10	7.796	257824	21.44	7200	
11	8.676	43470	3.62	2593	
12	9.005	115856	9.64	3282	
13	9.730	367576	30.57	25032	
14	13.531	149638	12.45	4431	


¦Â-asarone 40¦ÌL-1


	retention time	Peak area	Peak area(%)	Peak height	
1	0.439	15699	0.09	439	
2	2.643	14864	0.08	673	
3	4.144	27047	0.15	1569	
4	4.526	88709	0.49	7664	
5	5.056	30378	0.17	1341	
6	9.825	17796782	98.74	896996	
7	11.233	50302	0.28	2256	
